# Multi-breed genomic evaluation for tropical beef cattle when no pedigree information is available

**DOI:** 10.1186/s12711-023-00847-6

**Published:** 2023-10-16

**Authors:** Ben J. Hayes, James Copley, Elsie Dodd, Elizabeth M. Ross, Shannon Speight, Geoffry Fordyce

**Affiliations:** 1 Centre for Animal Science, Queensland Alliance for Agriculture and Food Innovation, University of Queensland, St Lucia, QLD 4067 Australia; 2 BlackBox Co, Mareeba, QLD 4880 Australia

## Abstract

**Background:**

It has been challenging to implement genomic selection in multi-breed tropical beef cattle populations. If commercial (often crossbred) animals could be used in the reference population for these genomic evaluations, this could allow for very large reference populations. In tropical beef systems, such animals often have no pedigree information. Here we investigate potential models for such data, using marker heterozygosity (to model heterosis) and breed composition derived from genetic markers, as covariates in the model. Models treated breed effects as either fixed or random, and included genomic best linear unbiased prediction (GBLUP) and BayesR. A tropically-adapted beef cattle dataset of 29,391 purebred, crossbred and composite commercial animals was used to evaluate the models.

**Results:**

Treating breed effects as random, in an approach analogous to genetic groups allowed partitioning of the genetic variance into within-breed and across breed-components (even with a large number of breeds), and estimation of within-breed and across-breed genomic estimated breeding values (GEBV). We demonstrate that moderately-accurate (0.30–0.43) GEBV can be calculated using these models. Treating breed effects as random gave more accurate GEBV than treating breed as fixed. A simple GBLUP model where no breed effects were fitted gave the same accuracy (and correlations of GEBV very close to 1) as a model where GEBV for within-breed and the GEBV for (random) across-breed effects were included. When GEBV were predicted for herds with no data in the reference population, BayesR resulted in the highest accuracy, with 3% accuracy improvement averaged across traits, especially when the validation population was less related to the reference population. Estimates of heterosis from our models were in line with previous estimates from beef cattle. A method for estimating the number of effective breed comparisons for each breed combination accumulated across contemporary groups is presented.

**Conclusions:**

When no pedigree is available, breed composition and heterosis for inclusion in multi-breed genomic evaluation can be estimated from genotypes. When GEBV were predicted for herds with no data in the reference population, BayesR resulted in the highest accuracy.

**Supplementary Information:**

The online version contains supplementary material available at 10.1186/s12711-023-00847-6.

## Background

Genomic selection has accelerated genetic gains in a number of livestock populations, particularly those based on a small number of breeds and in cases where large existing reference populations were available [1, 2]. The prime example of genomic selection is in Holstein and Jersey dairy cattle, for which semen samples from many progeny-tested bulls formed the basis of reference populations [3]. In other livestock populations, the implementation of genomic selection has proved more challenging, both because they consist of many different breeds and crossbreds, and because such ready-made historical reference populations that are available in dairy populations do not exist [4, 5].

Multi-breed genomic evaluations for livestock are desirable as they allow producers to select sires and dams across breeds, crossbreds and composites, which increase selection intensity. The advantage of multi-breed evaluations for increasing accuracy of genomic prediction, particularly for breeds with smaller reference set sizes, has been demonstrated in dairy cattle [6], sheep [4, 7] and beef cattle [8]. Several approaches for multi-breed genomic predictions have been proposed (see Misztal et al. [9] for a comprehensive review). de Roos et al. [10] made an assumption that the effects of single nucleotide polymorphisms (SNPs) would be shared across breeds if they were sufficiently dense. For this to occur, the causative mutations that affect a trait would have to be the same across breeds, and for multibreed predictions, SNPs would have to be close enough to the mutation so that the linkage disequilibrium phase between SNPs and the causative mutation persists across breeds. As a proxy for SNP–causative mutation linkage disequilibrium phase, they investigated the extent of SNP–SNP linkage disequilibrium across three *Bos taurus* breeds. They concluded that 300,000 equally-spaced SNPs would be sufficient for multi-breed genomic predictions. Goddard and Hayes [11] extended this analysis to more breeds. They also concluded that 300,000 SNPs would be sufficient for multibreed genomic predictions in *Bos taurus* breeds, but that the associations between causative mutations and SNPs were unlikely to persist across *Bos taurus* and *Bos indicus* breeds. With the wide-spread availability of whole-genome sequence data (including the 1000 bull genomes reference set [12]), which can be used to impute SNP datasets to whole-genome sequence, if the assumption that a causative mutation has the same effect across breeds is invoked, genomic prediction using these causative mutations should result in high accuracy genomic predictions from multi-breed evaluations. However only a small increase in multi-breed prediction accuracy has been observed using whole-genome sequence in cattle and sheep [13–16].

Kemper et al. [17] suggested that a Bayesian model with a prior that allowed SNPs to have moderate to large effects (e.g. BayesB [18] or BayesR [19]) may be more suitable for multibreed evaluations with high-density markers than best linear unbiased prediction (BLUP) models. Their reasoning was that these models might ascribe larger effects to individual SNPs, with effects possibly persisting across breeds, in contrast to BLUP models that “smear” effects of causal mutations across many SNPs on a large chromosome segment (as a result of the BLUP prior which is all SNPs have small, non-zero effects), which may not persist across breeds. Some evidence supporting this was provided from the analysis of a multi-breed dairy population [17].

In contrast to the models of de Roos [10] and Goddard and Hayes [11], a different approach for multi-breed evaluation is to allow for quantitative trait loci (QTL) with different effects, and/or different QTL segregating across breeds, by treating each breed as a different, but potentially correlated trait [20, 21]. These estimated genetic correlations from this approach indicate the extent of the QTL and their effects, shared across breeds.

Refining this type of model to specify the location of QTL that are different across breeds, the breed-of-origin of alleles (BOA) model [22] is based on the hypotheses that “in crossbred populations: (1) effects of SNPs may be breed-specific, and (2) linkage disequilibrium may not be restricted to markers that are tightly linked to the QTL”. They proposed a model for genomic selection to select for commercial crossbred performance with breed-specific effects of SNP alleles, i.e. the model with breed-specific allele effects (BSAM), which subsequently the authors have termed the BOA models [23]. Accuracies of genomic prediction from BOA models based on crossbred populations have been demonstrated to be more accurate than those from models that do not explicitly model the breed-of-origin of alleles, for some but not all traits [24].

The above models have all been implemented and evaluated in *Bos taurus* breeds. An even greater challenge is the analysis of data from cattle with both *Bos indicus* and *Bos taurus* ancestry, as these subspecies diverged 600,000 to 800,000 years ago [25]. Multi-subspecies populations are common in tropical beef production systems in Australia, Latin America and Indonesia, and in tropical dairy production systems in India and Africa, e.g. [26–28]. For the analysis of such data, Bolormaa et al. [29] suggested a model that is similar to the BOA model, but at the sub-species level. They suggested that allocating chromosome segments to *Bos indicus* or *Bos taurus* origin would take into account the fact, that given the long divergence time between the sub-species, the differences in allele frequencies at QTL would be large, resulting in substantially different SNP-QTL linkage disequilibrium patterns between the sub-species. For multi-sub-species populations, a model that attempts to capture the advantages of both the multi-breed models proposed by de Roos et al. [10], Kemper et al. [17] and the BOA models is the “multi-subspecies” model proposed by Warburton et al. [30]. In this BayesR model, which is inspired by the model of Bolormaa et al. [29], the sub-species of origin of the haplotypes is assigned, but within each sub-species it is assumed that high-density markers are in linkage disequilibrium with specific causative mutations in the sub-species.

Single-step BLUP (ssBLUP) which integrates pedigree, genomic and phenotype information is increasingly used in routine genomic evaluations. The metafounders’ model is a multi-breed extension of ssBLUP [31]. In the usual implementation of ssBLUP, pedigree founders are considered as unrelated. The metafounders’ approach assigns pedigree founders to an ancestral population (a breed or a line). The ancestral populations are represented as a "metafounder," a pseudo-individual included as a founder of the pedigree and similar to an “unknown parent group.” [32]. The metafounders may share a genetic relationship (as in the multi-trait models), which can be estimated from markers. It should be mentioned that none of the above multi-breed models have yet achieved high accuracies of prediction for animals of breeds that are not represented in the reference set, which suggests that more research is required in this area [9]. At present, the solution is to include at least some animals of all the target breed(s), and crossbreds, in the reference set.

Perhaps the largest multi-breed beef cattle routine genomic evaluations are conducted in Ireland by the Irish Cattle Breeding Federation (ICBF) [33]. The models in these evaluations fit breed proportion as a fixed effect, as a continuous variable (breed proportion for each animal) with a separate effect fitted in the models for each breed. Heterosis in crossbred animals was modelled from pedigree contributions of different breed sires and dams [34]. These evaluations include millions of animals. Efficient single-step methods have been used to combine pedigree, genomic and phenotype information for this population [35].

In practice, there are a number of challenges to overcome for multi-breed predictions, especially in tropical beef cattle where crosses of *Bos indicus* and *Bos taurus* cattle are common. In particular, multiple breeds must be measured in the same contemporary groups in order to disentangle contemporary group effects from breed effects. Using commercial data from purebred, crossbred and composite animals (rather than data collected on studs) in multi-breed evaluations is attractive in this regard, as the head-to-head comparison of chromosome segments from different breeds can be done on a large scale. Using commercial data in the reference set may also be attractive because large reference populations can be assembled relatively quickly, for example by collecting records from processing plants where carcass traits and intra-muscular fat are scored on every carcass or pregnancy test records for fertility. Commercial data are also recorded in the target environment for production, so that the genotype-by-environment interaction between stud and commercial environments is no longer an issue (e.g. [36]). However in tropical beef populations, often little is known about breed composition or the heterosis of these animals, and limited or no pedigree is available, so that models such as ssBLUP and metafounders cannot be readily applied.

Another challenge in analysing commercial animal data is that, particularly, in tropical environments, the genomes of individual animals can be composed of segments from many breeds, i.e. reflecting the need to balance productivity and adaptation, which results in the use and testing of many breeds in these systems over time. With large numbers of breeds, some of the above models may be computationally challenging; for example the incidence matrices in the BOA models expand to SNPs $\times$ number of breeds.

Here, we propose simple models for multi-breed genomic prediction based on large-scale commercial data that overcome at least some of these problems. One of the models treats breeds as analogous to genetic groups [32], which allows partition of the genetic variance into within-breed and between-breed components, even if a large number of breeds is involved. Our method is similar to the ‘genomic groups’ approach described by Plieschke et al. [37], which is extended to account for crossbred and composite animals from a large number of breeds.

## Methods

### Animals, phenotypes and genotypes

Fifty-four collaborator beef cattle herds from across tropical Australia participated in data collection for the Northern Genomics project. The properties were located across Northern Australia, including South Western, Central, Western and Northern Queenslandand the Northern Territory of Australia, and the Pilbara region of Western Australia [38]. These regions are characterised by a wet season, and a dry season with little pasture growth. Central and South Western Queensland regions tend to have good to high quality pasture, while Western Queensland and Western Australia regions are arid with low rainfall (350 mm or less per year) [38]. The 29,321 heifers enrolled included crossbred and approximately 8000 purebred heifers from at least 14 breeds, i.e. Angus, Belmont Red, Brahman, Charolais, Droughtmaster, Hereford, Limousin, MurrayGrey, SantaGertrudis, Shorthorn, Wagyu, Boran, Senepol, Tuli (for genetic distances and diversity parameters of these breeds see [39]).

Importantly, producers in this project are committed to have bulls in the paddock for at least 6 weeks. Traits measured from 2015 to 2020 on the heifers included live weight, hip height and body condition score (BCS) at an average of 600 days, and a heifer puberty trait. The heifer puberty trait was cycling or not cycling by an average of 600 days (heifer puberty) assessed by the presence or absence of *corpus luteum* using ovary scanning [40]. To maximise genetic variation, the trait is actually measured when an estimated (by average live weight) 50% of the heifers are pubertal, i.e., at 1 to 2.5 years of age. Further details on the phenotypes are described in Copley et al. [38].

All heifers were genotyped with the 35 k or 50 k TropBeef SNP array by Neogen, Australasia. SNPs were removed if more than 10% of the genotypes were missing for that SNP. If an individual genotype had a GC score less 0.6, it was set to missing and recovered by imputation. Genotypes were imputed up to 709,768 SNPs (bovine high-density (HD) array) using the findhap software [41] and a panel of 4506 cattle from relevant breeds that were genotyped with the Bovine HD array. This panel of HD SNPs was obtained by removing the SNPs that had less than 10 copies of the minor allele in the imputation reference panel, and the SNPs that had more than 10% missing genotypes. All heifer breeds were represented by at least 50 animals that were genotyped with the Bovine HD array in the imputation reference set. The accuracy of imputation was at least 93% for all breeds/crossbreds.

### Statistical models

**Fig. 1:**
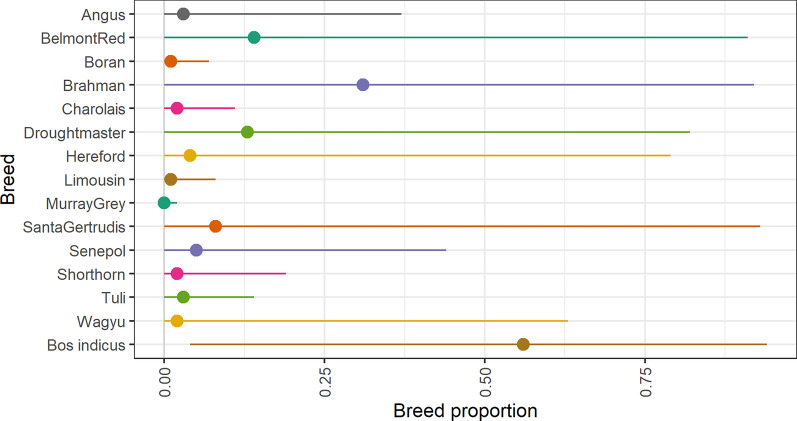
Average proportion of each breed, and *Bos indicus* proportion, across the 54 herds. Average proportion of each breed, maximum and minimum average proportions for each of the herds

First, we estimated the breed proportions of each heifer for each of the 14 breeds known to be in the dataset using the 35 K array data only (Fig. 1). A separate large dataset consisting of 4506 purebred cattle was used to estimate SNP effects for breed composition. This dataset included at least 50 cattle from all the breeds that comprised the heifers’ ancestry. A genomic (G)BLUP model was fitted, where the phenotype was 1 if the animal was from that breed and 0 if not [42, 43]. The effect of each SNP for the proportion of each breed was then derived by back-solving for the SNP effects, treating breed as a trait [44], and the resulting prediction equations for each breed were used to estimate the breed proportions in the heifers.

To predict genomic estimated breeding values (GEBV), four multi-breed models were evaluated.

- Model 1 (BREEDFIXED) was:
  $$\mathbf{y}=\mathbf{1n}\upmu +\mathbf{X}\mathbf{f}+\mathbf{W}\mathbf{b}+\mathbf{Z}\mathbf{u}+{\varvec{\upvarepsilon}},$$

where $\mathbf{y}$ is the vector of trait records (Clscore, live weight, hip height or body condition score); $\upmu$ is the population mean, and $\mathbf{1n}$ is a vector of 1s; $\mathbf{f}$ is the vector of fixed effects including contemporary group, the herd + birth year + paddock (enclosed field) that the heifers were in before they were gathered together for trait recording (there were 148 contemporary groups, each group included at least 50 animals), the year of recording (2015, 2016, 2017, 2018, 2019, and 2020), and $\mathbf{f}$ also included a linear covariate of the heterozygosity of each heifer as measured by the proportion of marker genotypes that were heterozygous (to capture heterosis effects); $\mathbf{X}$ is the design matrix that relates fixed effects to records; $\mathbf{W}$ is the matrix of records $\times$ number of breeds, with each element, measuring the proportion of each breed in the heifers as described above (14 breeds); $\mathbf{b}$ is the vector of fixed effects for each of the 14 breeds; $\mathbf{u}$ is the vector of random genetic effects ~ $N(\mathbf{0},\mathbf{G}{\sigma }_{g}^{2})$ with $\mathbf{G}$ being the genomic relationship matrix between all the heifers and ${\sigma }_{g}^{2}$ the genetic variance captured by the SNPs, with $\mathbf{G}$ constructed according to method 1 of [45]; $\mathbf{Z}$ is the design matrix that relates records to animals; ${\varvec{\upvarepsilon}}$ is the vector of random deviations ~ $N(\mathbf{0}, \mathbf{I}{\sigma }_{e}^{2})$ with $\mathbf{I}$ an animal-by-animal identity matrix and ${\sigma }_{e}^{2}$ is the error variance.

There were at least 50 animals in each contemporary group. Marker heterozygosity in the dataset ranged from ~ 0.25 (for a purebred) to 0.5 (for an F1 cross). Maximum heterosis was achieved for F1 crosses (e.g. when marker heterozygosity was 0.5) and minimum heterosis was achieved for purebreds (when marker heterozygosity was 0.25). Accordingly, we re-scaled the estimate of heterosis to be on the scale of purebred (0) to F1 (1). For this model, variance components were estimated with the genome-wide complex trait analysis (GCTA) tool [44], using the greml option with separate files for fixed effects (covar) and covariates (qcovar), and the heritability of the traits (in this case, the proportion of phenotypic variance captured by the SNPs) was estimated as ${h}^{2}=\widehat{{\sigma }_{g}^{2}}/(\widehat{{\sigma }_{g}^{2}}+\widehat{{\sigma }_{e}^{2}}).$ The GEBV for this model were predicted as: $\mathbf{G}\mathbf{E}\mathbf{B}\mathbf{V}=\mathbf{W}\widehat{\mathbf{b}}+\widehat{\mathbf{u}}$.

- Model 2 (BREEDRANDOM) was:
  $$\mathbf{y}=\mathbf{1n}\upmu +\mathbf{X}\mathbf{f}+\mathbf{Z}\mathbf{a}+\mathbf{Z}\mathbf{u}+{\varvec{\upvarepsilon}},$$

where effects are defined as above, and $\mathbf{a}$ is a vector of random variables (the breed effects combined into one value for an animal) and follows ~ $N(\mathbf{0},\mathbf{B}{\sigma }_{b}^{2})$, where $\mathbf{B}=\mathbf{W}{\mathbf{W}}^{\mathbf{T}}$. A small amount (0.01) was added to the diagonal to aid with matrix inversion. If desired, the estimates of breed effects can be obtained from this model using the following derivation. If we assume that the combined across-breed breeding value, or more precisely the effect pertaining to a particular breed compisition ($\mathbf{a}$) is a weighted sum of (the now random) individual breed effects ($\mathbf{b}$), then the estimates of breed effects can be obtained as follows:

Noting that $\widehat{\mathbf{a}}=\mathbf{W}\widehat{\mathbf{b}}$, multiplying both sides by ${\mathbf{W}}^{\mathbf{T}}$ results in ${\mathbf{W}}^{\mathbf{T}}\widehat{\mathbf{a}}={\mathbf{W}}^{\mathbf{T}}\mathbf{W}\widehat{\mathbf{b}}$. Then, multiplying both sides by the inverse of ${\mathbf{W}}^{\mathbf{T}}\mathbf{W}$ we obtain ${{\left[{\mathbf{W}}^{\mathbf{T}}\mathbf{W}\right]}^{-1}\mathbf{W}}^{\mathbf{T}}\widehat{\mathbf{a}}={{\left[{\mathbf{W}}^{\mathbf{T}}\mathbf{W}\right]}^{-1}\mathbf{W}}^{\mathbf{T}}\mathbf{W}\widehat{\mathbf{b}}$, such that the estimates of breed effects equal $\widehat{\mathbf{b}}={{\left[{\mathbf{W}}^{\mathbf{T}}\mathbf{W}\right]}^{-1}\mathbf{W}}^{\mathbf{T}}\widehat{\mathbf{a}}$.

For this model, $\mathbf{G}\mathbf{E}\mathbf{B}\mathbf{V}=\widehat{\mathbf{a}}+\widehat{\mathbf{u}}$.

- Model 3 (NOBREED)

A GBLUP model was also fitted where no breed effects were fitted, in order to investigate if the GEBV from this model were the same as $\widehat{\mathbf{a}}+\widehat{\mathbf{u}}$ (i.e. within- and across-breed estimates of the breeding values from the BREEDRANDOM model). The model for NOBREED was:

$$\mathbf{y}=\mathbf{1n}\upmu +\mathbf{X}\mathbf{f}+\mathbf{Z}\mathbf{u}+{\varvec{\upvarepsilon}},$$

where terms are as above.

For this model $\mathbf{G}\mathbf{E}\mathbf{B}\mathbf{V}=\widehat{\mathbf{u}}$.

- Model 4 (BAYESR)

Finally a BayesR model was fitted (BAYESR):

$$\mathbf{y}=\mathbf{1n}\upmu +\mathbf{X}\mathbf{f}+\mathbf{S}\mathbf{g}+{\varvec{\upvarepsilon}},$$

where effects are as above. $\mathbf{S}$ is the matrix of records by animal allocating genotypes to records, where the genotypes were recorded as 0 = *AA*, 1 = *AB*, 2 = *BB*, and in the matrix $\mathbf{S}$, these genotypes were centred and standardized; $\mathbf{g}$ is a vector of SNP effects. In BayesR, the variance associated with the i^th^ SNP is assumed to come from one of four distributions, either ${\upsigma }_{\mathrm{i}}^{2}=0$, or ${10}^{-4}{\upsigma }_{\mathrm{g}}^{2}$, or ${10}^{-3}{\upsigma }_{\mathrm{g}}^{2}$, or ${{10}^{-2}\upsigma }_{\mathrm{g}}^{2}$; where ${\upsigma }_{\mathrm{g}}^{2}$ is the genetic variance of the trait. This allows the BayesR model [19] to have a flexible SNP effect distribution, which is a mixture of four possible normal distributions, either $N\left(\mathrm{0,0}\right)$ or $N\left(0,{ 10}^{-4}{\upsigma }_{\mathrm{g}}^{2}\right)$, or ${N(\mathrm{0,10}}^{-3}{\upsigma }_{\mathrm{g}}^{2})$, or ${N(0,{10}^{-2}\upsigma }_{\mathrm{g})}^{2}$, all with a mean of 0 but with different variances. Note that no breed effects were fitted in this model.

For the BAYESR model, $\mathbf{G}\mathbf{E}\mathbf{B}\mathbf{V}=\mathbf{S}\widehat{\mathbf{g}}$.

### Validation

The accuracy of GEBV from each of the four models was evaluated by selecting at random 10 herds for validation, and then for each herd dropping the last contemporary group (the latest birth year of heifers for which data were recorded) for validation. The breed composition of these validation contemporary groups (in total, 3790 heifers) ranged from purebred Brahman to crossbreds of *Bos taurus* breeds. Genomic estimated breeding values were predicted for the heifers in the 10 excluded contemporary groups, then the GEBV were correlated with the actual phenotypes (adjusted for fixed effects) of the heifers within each contemporary group, and then averaged over the 10 groups. This correlation was divided by the square root of the heritability of the trait to obtain the accuracy of genomic prediction. Validations were also performed when the data for the entire herd for each of the 10 randomly chosen herds was removed. Genetic parameters for all the models were re-estimated when the validation sets were removed.

We also assessed the accuracy of genomic predictions for heifer puberty in a completely independent dataset, i.e. the Beef CRC groups of 894 and 1088 Brahman and Tropical composite cattle (respectively). These cattle were phenotyped for a different, but related fertility trait, than the one we used, which was age at first *corpus luteum*, assessed by ultrasound scanning at six-week intervals [46]. Genotypes for these cattle were imputed to the same 709,768 SNPs used in the genomic predictions.

Ultimately for multi-breed evaluations, head-to-head comparisons of breeds in the same herd/environment are necessary. We assessed the number of head-to-head comparisons as $\sum_{i}^{n}{\mathbf{W}}_{{\varvec{i}}}^{\boldsymbol{^{\prime}}}{\mathbf{W}}_{{\varvec{i}}}$, where for each of the $n$ contemporary groups in the dataset, ${\mathbf{W}}_{i}$ is a matrix of breed proportions in contemporary group $i$, of dimensions equal to the number of heifers in the contemporary group $\times$ number of breeds. This formula sums the number of head-to-head comparisons made within each contemporary group, then for each breed-by-breed comparison sums across contemporary groups. Standard errors on breed-by-breed comparisons can also be calculated, and these reflect the number of head-to-head comparisons within contemporary groups. An example of standard errors is given for the Angus comparisons (see Additional file 1: Table S1).

## Results

### Breed composition of the reference population

The average proportion of each breed across herds, and the maximum proportion and minimum proportion of each breed across herds are given in Fig. 1. The error of the prediction of breed proportions was derived by calculating the prediction for an independent dataset of animals of known breed; this error was 0.05.

### Estimated heterosis and breed effects

Estimates of heterosis were similar across all models (Table 1) although slightly smaller for BAYESR, perhaps because this model captures more additive variance (Table 2). Heterosis was substantial for some traits such as weight and heifer puberty, and was positive for all traits (Table 1).

**Table 1:** Estimates of heterosis for tropically-adapted beef heifer traits at approximately 600 days of age from four multi-breed models

	Live weight (kg)	Hip height (mm)	Body condition score (1–5)	Heifer puberty (0 or 1)
Number of records	26,721	25,567	26,794	29,367
BREEDFIXED	47.6 ± 2.9	50.5 ± 3.8	0.30 ± 0.03	0.47 ± 0.03
BREEDRANDOM	45.4 ± 2.8	48.4 ± 3.7	0.30 ± 0.03	0.44 ± 0.03
NOBREED	47.0 ± 2.9	50.5 ± 3.7	0.30 ± 0.03	0.46 ± 0.03
BAYESR	40.6 ± 2.8	46.3 ± 4.3	0.22 ± 0.03	0.46 ± 0.05

**Table 2:** Variance components and heritability estimates from four multi-breed models for tropically-adapted beef heifer traits at approximately 600 days of age

	Number of records	BREEDFIXED		BREEDRANDOM			NOBREED		BAYESR	
		Vg	Ve	Vg_w	Vg_b	Ve	Vg	Ve	Vg	Ve
Variance components*										
Live weight (kg)	26,721	455.7 ± 19.0	1006.9 ± 14.7	444.5 ± 19.0	367.8 ± 190.4	1027.7 ± 14.7	455.5 ± 19.0	1022.8 ± 14.7	461.1 ± 19.0	1006.1 ± 16.6
Hip height (mm)	25,567	923.5 ± 33.7	1393.9 ± 23.0	960.5 ± 22.2	668.7 ± 339.8	1341.8 ± 33.2	969.4 ± 33.9	1407.2 ± 22.9	920.2 ± 30.0	1447.8 ± 26.6
Body condition score	26,794	0.03 ± 0.001	0.10 ± 0.001	0.03 ± 0.001	0.003 ± 0.003	0.10 ± 0.001	0.03 ± 0.001	0.10 ± 0.001	0.03 ± 0.001	0.10 ± 0.001
Heifer puberty	29,367	0.05 ± 0.002	0.16 ± 0.002	0.05 ± 0.002	0.02 ± 0.014	0.10 ± 0.014	0.05 ± 0.002	0.16 ± 0.002	0.05 ± 0.002	0.15 ± 0.002
*Heritabilities*										
Live weight (kg)	26,721	0.30 ± 0.01		0.24 ± 0.03	0.20 ± 0.08		0.31 ± 0.01		0.31 ± 0.01	
Hip height (mm)	25,567	0.40 ± 0.01		0.32 ± 0.04	0.23 ± 0.09		0.41 ± 0.01		0.39 ± 0.01	
Body condition score	26,794	0.22 ± 0.01		0.22 ± 0.01	0.02 ± 0.02		0.22 ± 0.01		0.24 ± 0.01	
Heifer puberty	29,367	0.22 ± 0.01		0.20 ± 0.02	0.10 ± 0.05		0.23 ± 0.01		0.25 ± 0.01	

Variance components are the genetic vairance, Vg and Ve the error variance. In addition, the BREEDRANDOM model estimates within-breed (Vg_w) and across-breed genetic variance components (Vg_b)

**Fig. 2:**
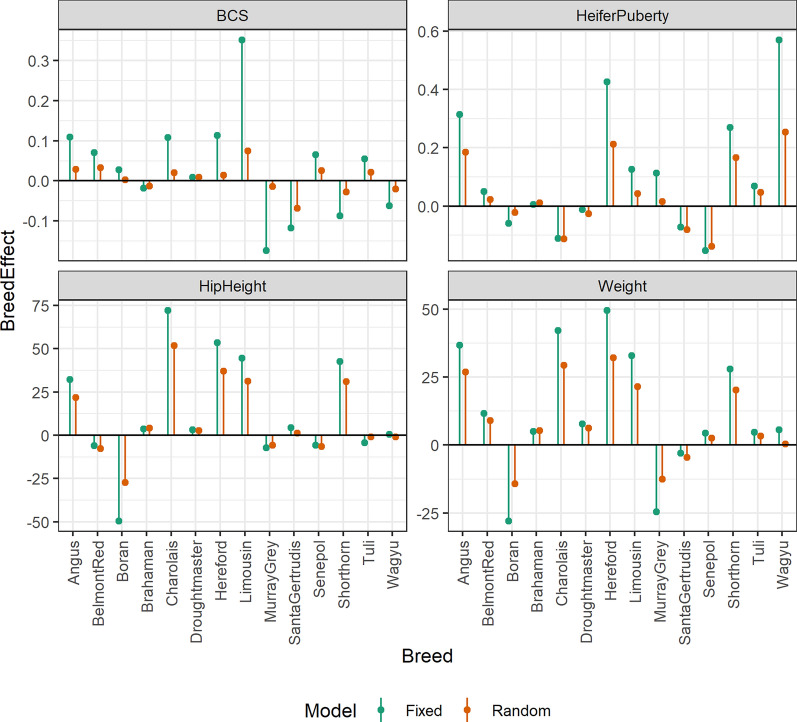
Estimates of breed effects. Estimates of breed effects for body condition score (BCS), heifer puberty, liveweight and hip height when effects were treated as either fixed (BREEDFIXED model) or random (BREEDRANDOM)

Breed effects across models were similar for the breeds that were well represented in the dataset. However, for breeds such as Tuli, Wagyu and Senepol, for which only a few animals were available thus representing a moderate to small proportion of the breeds, the estimated effects were much smaller than for the other breeds when effects were treated as random, reflecting substantial shrinkage towards 0 (Fig. 2). The correlation between the random and fixed effect breed estimates was above 0.9 for all traits.

### Genetic parameter estimates

Estimates of heritability from the genomic data were quite similar from all four models (Table 2) although the NOBREED and BAYESR models gave slightly higher estimates, which might be expected as breed effects were not explicitly fitted in these models. The estimated heritability was moderate for heifer puberty and body condition score, and higher for live weight and hip height (Table 2).

For the BREEDRANDOM model, the within-breed and across-breed variance were almost equal for weight, while almost all the variance was within-breed for body condition score, and split into two thirds and one third, respectively, for heifer puberty condition score.

### Accuracies of GEBV for the validation set

The correlations between the GEBV from the different models for each trait are in Table 3. The lowest correlations were between the BREEDFIXED and BAYESR models. Model BREEDRANDOM and NOBREED gave very similar GEBV for all traits, suggesting these models are almost equivalent at the level of GEBV. The BREEDFIXED model produced GEBV that were consistently greater in magnitude than the models treating breed as random, which probably reflects the fact that breed effects could be over-dispersed with this model.

**Table 3:** Correlations between the GEBV from the different models for four traits

Models compared	Live weight	Hip height	BCS	Heifer puberty
*Correlation*				
BREEDFIXED – BREEDRANDOM	0.97	0.96	0.96	0.98
BREEDFIXED – NOBREED	0.96	0.94	0.95	0.97
BREEDFIXED – BAYESR	0.93	0.90	0.92	0.95
BREEDRANDOM – NOBREED	1.00	0.98	1.00	1.00
BREEDRANDOM – BAYESR	0.97	0.95	0.98	0.98
NOBREED – BAYESR	0.97	0.96	0.98	0.98
*Regression (first model GEBV on second model GEBV)*				
BREEDFIXED – BREEDRANDOM	1.21	1.01	1.06	1.20
BREEDFIXED – NOBREED	1.21	1.00	1.06	1.21
BREEDFIXED – BAYESR	1.21	1.02	1.01	1.20
BREEDRANDOM – NOBREED	1.01	1.00	1.00	1.02
BREEDRANDOM – BAYESR	1.01	1.03	0.97	1.00
NOBREED – BAYESR	1.00	1.01	0.96	0.99

Standard errors for the correlations are 0.006, using Fischer’s approximation of 1/√(n-3)

BCS = body condition score

**Fig. 3:**
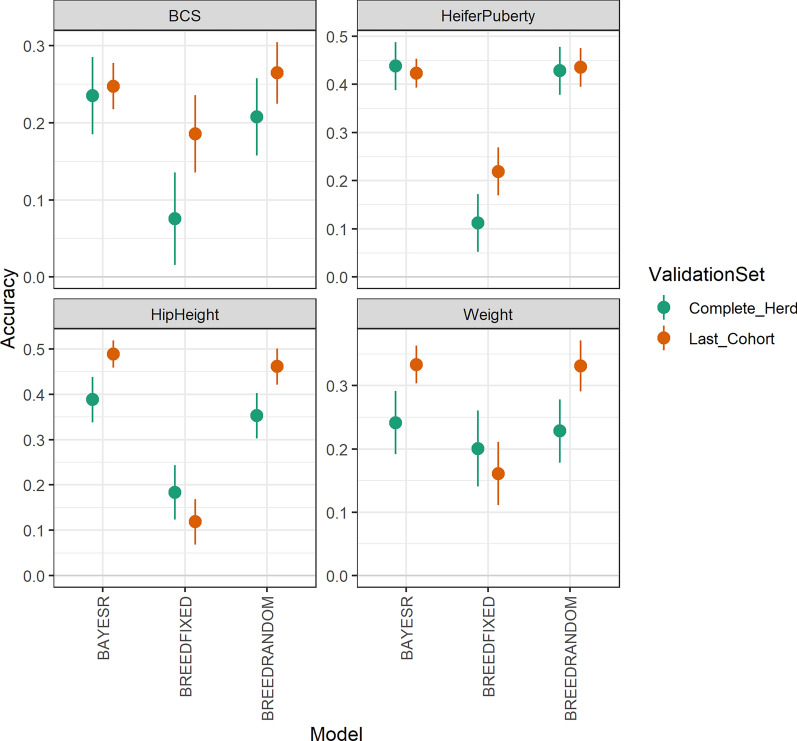
Accuracies of GEBV from three prediction methods (BREEDFIXED, BREEDRANDOM and BAYESR). The validation set consisted of 10 herds, with all data from these 10 herds completely removed, or only the last contemporary group of data from each contemporary group removed from the reference set

Accuracies of GEBV in the 10 validation contemporary groups were moderate, and fairly consistent across herds, which is reflected by the relatively small standard error of these accuracies (Fig. 3). BREEDRANDOM gave considerably higher accuracies than BREEDFIXED, for all traits, regardless of whether the validation was with all contemporary groups in the 10 validation herds completely removed from the reference set, or if only the last contemporary group of data was removed. BAYESR gave slightly higher accuracies, for all traits, when the validation was for all data for the removed 10 validation herds but was similar to BREEDRANDOM when the validation was for the last contemporary group of data from the removed validation herds.

In the Beef CRC validation, the correlation between the GEBV for age at corpus luteum and heifer puberty was − 0.45 for 894 Brahmans and − 0.21 for 1088 Tropical composites. This negative correlation is expected, as a younger age at corpus luetum should be associated with a higher probability that the heifer has a corpus luteum at 600 days. The stronger negative correlation in the Brahmans than in the Tropical composites, may reflect the substantial representation of Brahmans in the reference population.

The number of head-to-head comparisons that were possible from the dataset and allowed estimation of the breed effects, was largest for Angus versus Brahman (337), Brahman versus Droughtmaster (924) and Brahman versus Santa Gertrudis (421), but smallest for Wagyu and Belmont Red (Table 4).

**Table 4:** Number of head-to-head breed comparisons, where each cell represents the number of genomes for a breed being compared to the number of genomes of the other breed

	Angus	Belmont Red	Brahman	Charolais	Droughtmaster	Hereford	Limousin	Santa Gertrudis	Shorthorn	Wagyu
Belmont Red	7									
Brahman	337	24								
Charolais	20	2	153							
Droughtmaster	123	15	924	44						
Hereford	47	4	76	12	58					
Limousin	21	3	97	13	33	13				
Santa Gertrudis	111	7	421	31	208	35	27			
Shorthorn	33	3	73	10	61	13	10	44		
Wagyu	3	0	14	2	16	2	3	8	2	
Boran	2	4	33	1	7	1	1	3	2	0

Murray Grey, Tuli, Senepol are omitted from the Table as they represent a very small proportion of the reference population

## Discussion

This study demonstrates that moderate accuracies of multi-breed genomic prediction can be achieved from large-scale commercial tropical beef reference datasets. Genomic heritabilities were consistent with previous estimates based on pedigree data for these traits for tropically-adapted beef cattle [40], as well as the heterotic effects [46] and breed effects [47-49].

In multi-breed commercial data, the challenges of unknown breed composition and level of heterosis can be solved using estimates of these parameters that are obtained directly from the genotype data, as previously demonstrated in both cattle and pigs [50–53], provided there is a reference population of purebred genotypes. Using crossbred data allows head-to-head comparison of chromosome segments derived from different breeds, which allows separation of breed effects from contemporary group effects, at least to some extent (Fig. 2).

When cattle are derived from many breeds, and particularly when some of these breeds are represented by only a small number of individuals or only as a small proportion of the genome of a limited number of animals, treating breeds as a random effect rather than a fixed effect appears to result in more accurate multi-breed genomic evaluations. This is likely because when breeds are treated as a fixed effect, the effects for breeds with limited representation in the dataset are over-estimated in magnitude, which reduces the accuracy of the multi-breed GEBV.

The BREEDRANDOM model is attractive in that it results in two GEBV, a within-breed GEBV and a (single) across-breed GEBV, and it also partitions the genetic variance into within-breed and across-breed components. This may be useful for comparisons with existing within-breed evaluations, and for understanding how genetic variation is portioned within and between breeds. However, care should be taken when implementing this model; for example, if breeds are nested within environments, the breed effect will capture the environment effect as well, resulting in erroneous genomic evaluations (note that this will affect other models as well). The ${\sigma }_{b}^{2}$ in the BREEDRANDOM model is the variation in the population due to the different means for the traits for different breeds, which in turn are a result of either different QTL segregating in different breeds, the same QTL segregating across breeds at different frequencies, as well as different linkage disequilibrium patterns between SNP sand QTL in the different breeds. The differences across traits that we observed in the partitioning of the within-breed and between-breed variances is likely to be, at least partly, due to different QTL segregating in the *Bos indicus* and *Bos taurus* populations [29, 30].

If the allele frequencies in the base population for each breed were calculated, and further corrected to represent an F1 cross for each breed, ${\sigma }_{b}^{2}$ would then reflect the variance in the base population from which all breeds diverged [19, 54]. This would be an interesting approach and requires further investigation.

Fitting a separate random effect for breed, and allowing the genomic relationship matrix to capture breed effects (e.g. BREEDRANDOM and NOBREED models) resulted in very similar GEBV. The NOBREED model may be easier to implement as only one genomic relationship matrix is included in the model; however the NOBREED model does not partition the within- or across-breed genetic variance. Plieschke et al. [37] made a similar observation with both their genetic group model and their standard GBLUP, which were similar to our BREEDRANDOM and NOBREED models, respectively.

Genomic predictions from BAYESR were slightly more accurate for all traits than those from BREEDRANDOM, when the validation set included entire herds, but accuracies from the two models were very similar when the validation set included only the last contemporary group of animals from the 10 validation herds. This is likely because when the reference population includes data from the validation herds (other contemporary groups, except for the most recent one, from the validation herds were included in the reference set), there are some substantial genomic relationships between the reference and the validation sets (e.g. half sibs, quarter sibs), and GBLUP can take advantage of these for quite accurate prediction. When entire herds are dropped out, the relationships between the reference and validation sets are more distant and prediction is based more on individual SNP effects, which favours BAYESR.

One issue with the BREEDRANDOM and NOBREED models described here is that as breed proportions in the dataset change over time (as more animals are genotyped), the $\mathbf{G}$ matrix will be centred and scaled differently, such that variance components will be data-set dependent. REML updates of variance components will be required for each situation. This will have to be pointed out to the users of the GEBV. However, since no pedigree is used, we do not have the problem of the $\mathbf{A}$ and $\mathbf{G}$ matrices being on different bases, which may occur in a single step analysis, e.g. [9].

It is important to note that, in theory, models used to predict (and properly define) estimated breeding values in crossbred populations should allow different (additive) variances in each purebred population, as well as in each crossbred population [55–57]. We have not taken this approach here, because the size and structure of our dataset are unlikely to support the estimation of 14 within-breed variances as well as variances for each breed $\times$ breed interactions. Nevertheless, as the dataset gets larger, it may be worth attempting a more precise partitioning of the variance to assess if higher accuracies of prediction could be achieved with such models.

## Conclusions

In conclusion, data from crossbred and composite commercial animals (with no pedigree) can be used to construct reference populations for genomic prediction, provided the data collected are of high quality (e.g., consistent measurement protocols, accurate matching of IDs and records). Such data could be collected on a very large scale, for example for carcass traits from processing plants, and for some fertility traits. Breed composition of animals can be recovered from the marker genotypes (provided there is a reference population of pure-bred animal genotypes), as marker heterozygosity which can be used as a proxy for heterosis. Extensive head-to-head comparisons of chromosome segments from different breeds in the same contemporary group are necessary for accurate multi-breed genomic evaluations, and the extent of these comparisons in a dataset can be determined from the genomic data.

### Supplementary Information

**Additional file 1: Table S1.** Examples of standard errors of breed contrasts for weight (kg).

## Data Availability

Not applicable.
