# Supplementary material for: Multi-breed genomic evaluation for tropical beef cattle when no pedigree information is available
Source: Genet Sel Evol. 2023 Oct 16;55:71. doi: 10.1186/s12711-023-00847-6 (PMC10578004; doi:10.1186/s12711-023-00847-6)
Supplement: Supplementary file 1 — Additional file 1: Table S1. Examples of standard errors of breed contrasts for weight (kg). [file 12711_2023_847_MOESM1_ESM.docx]

**Table S1. Examples of Standard errors of breed contrasts for weight (kg).**

| **Breed contrasts** | **Standard error** |
| --- | --- |
| Angus:BelmontRed | 43.9 |
| Angus:Brahaman | 18.9 |
| Angus:Charolais | 94.6 |
| Angus:Droughtmaster | 30.1 |
| Angus:Hereford | 47.7 |
| Angus:Limousin | 95.2 |
| Angus:MurrayGrey | 145.8 |
| Angus:SantaGertrudis | 24.6 |
